# Screening of inmates transferred to Spain reveals a Peruvian prison as a reservoir of persistent *Mycobacterium tuberculosis* MDR strains and mixed infections

**DOI:** 10.1038/s41598-020-59373-w

**Published:** 2020-02-17

**Authors:** Estefanía Abascal, Marta Herranz, Fermín Acosta, Juan Agapito, Andrea M. Cabibbe, Johana Monteserin, María Jesús Ruiz Serrano, Paloma Gijón, Francisco Fernández-González, Nuria Lozano, Álvaro Chiner-Oms, Tatiana Cáceres, Pilar Gómez Pintado, Enrique Acín, Eddy Valencia, Patricia Muñoz, Iñaki Comas, Daniela M. Cirillo, Viviana Ritacco, Eduardo Gotuzzo, Darío García de Viedma

**Affiliations:** 1 Hospital General Universitario Gregorio Marañón. Instituto de Investigación Sanitaria Gregorio Marañón, Madrid, Spain; 2 CIBER Enfermedades Respiratorias, (CIBERES), Spain; 3 TB Research Unit, Instituto de Medicina Tropical Alexander von Humboldt, Universidad Peruana Cayetano Heredia, Lima, Peru; 4 Emerging Bacterial Pathogens Unit, IRCCS San Raffaele Scientific Institute, Milan, Italy; 5 Instituto Nacional de Enfermedades Infecciosas INEI-ANLIS, Ciudad Autónoma de Buenos Aires, Buenos Aires, Argentina; 6 Consejo Nacional de Investigaciones Científicas y Técnicas CONICET, Ciudad Autónoma de Buenos Aires, Buenos Aires, Argentina; 7 Unidad Mixta Genómica y Salud, Centro Superior de Investigación en Salud Pública (FISABIO)-Universitat de València, Valencia, Spain; 8 General Subdirection of Penitentiary Health – Penitentiary Institutions – Ministry of Interior of Spain, Madrid, Spain; 9 Instituto Nacional de Salud, Lima, Peru; 10 Instituto de Biomedicina de Valencia (IBV) Consejo Superior de Investigaciones Científicas (CSIC), Valencia, Spain; 11 CIBER en Epidemiología y Salud, Pública, Spain

**Keywords:** Clinical microbiology, Epidemiology

## Abstract

It is relevant to evaluate MDR-tuberculosis in prisons and its impact on the global epidemiology of this disease. However, systematic molecular epidemiology programs in prisons are lacking. A health-screening program performed on arrival for inmates transferred from Peruvian prisons to Spain led to the diagnosis of five MDR-TB cases from one of the biggest prisons in Latin America. They grouped into two MIRU-VNTR–clusters (Callao-1 and Callao-2), suggesting a reservoir of two prevalent MDR strains. A high-rate of overexposure was deduced because one of the five cases was coinfected by a pansusceptible strain. Callao-1 strain was also identified in 2018 in a community case in Spain who had been in the same Peruvian prison in 2002–5. A strain-specific-PCR tailored from WGS data was implemented in Peru, allowing the confirmation that these strains were currently responsible for the majority of the MDR cases in that prison, including a new mixed infection.

## Introduction

Certain types of tuberculosis (TB) are diagnostically and therapeutically challenging. Examples include infection by multidrug-resistant strains of *Mycobacterium tuberculosis* (MDR-TB, resistant at least to isoniazid and rifampicin) and mixed infections by more than one strain, especially when resistant and susceptible strains are involved. Both MDR-TB and mixed infections were initially considered to be restricted to high-burden settings without suitable TB control programs. However, international migratory movements make it very likely that they will also be found in low-burden settings.

Identification of hot-spots for the transmission of MDR strains is increasingly easy thanks to molecular epidemiology strategies, which have recently improved with the application of whole genome sequencing (WGS). In these settings, also a higher rate of mixed infections could be found, even involving resistant and susceptible strains, most likely due to re-exposure to infectious cases. However, systematic fingerprinting and genomic analysis are not generally performed in the high-burden settings where these kind of infections are expected to be more frequent.

Cooperative multinational efforts provide an opportunity to offset local limitations. Joint studies revealed settings with active transmission of MDR-TB^1–3^ and made it possible to evaluate the impact on host countries of the distribution of resistant strains introduced by migrant TB cases, which are circulating in their countries of origin^4,5^.

In this study, we formed a multinational consortium to identify the highly complex situation of TB in a large Peruvian prison, where multiresistance overlaps with mixed infections. We followed an indirect approach, by analysing Spanish inmates with TB who were transferred from the Peruvian prison to a Spanish prison to complete their sentences. In addition, we expanded the analysis beyond the prisoners to the general population and to migrant Peruvian populations in Spain, Italy, and Argentina. In order to survey one of the prison strains, we implemented an optimized strategy consisting of a specific PCR targeting strain-marker SNPs (Single Nucleotide Polymorphism) identified from WGS data. This approach provided a more precise snapshot of the situation both in the country of origin and in the host countries receiving the cases, and thus paved the way for more efficient interventions.

## Methods

### Screening transferred inmates for TB

Since 2016, 34 inmates transferred from Peruvian prisons to Spanish prisons in Madrid have been screened to detect TB infection. The microbiological determination involved three sputa from each prisoner taken at reception and processed according to standard methods, including decontamination and culture in Löwenstein–Jensen and MGIT media (Mycobacterial Growth Indicator tube; Becton Dickinson, Sparks, Maryland, USA). A drug sensitivity test was also performed for positive cultures using phenotypic susceptibility testing (SIRE (S: streptomycin, I: isoniazid, R: rifampicin, E: ethambutol) and PZA (pyrazinamide); applying the standard critical concentrations to detect resistances: 1.0 μg/ml for S, 0.4 μg/ml for I, 1.0 μg/ml for R, 5.0 μg/ml for E and 100 μg/ml for PZA; BACTEC™ MGIT™ 960 SIRE Kit and BACTEC™ MGIT™ 960 PZA Kit, Becton Dickinson, Sparks, Maryland, USA). Genotypic tests (Xpert® MTB/RIF; Cepheid, Sunnyvale, USA) to identify resistance mutations were additionally performed. All these procedures were perfomed following the biosafety measures required for handling *M. tuberculosis* (MTB) in BSL-3 laboratories.

### MIRU-VNTR analysis

All MTB isolates were fingerprinted by 24-Locus MIRU-VNTR as described elsewhere^6^. Briefly, DNA was extracted and purified from positive cultures by using a column-based method (QIAamp DNA minikit; Qiagen, Courtaboeuf, France) according to the manufacturer’s protocol. To accomplish biosafety requirements, the first extraction steps were performed in BSL-3 laboratories, moving to BSL-2 laboratories once mycobacterias were inactivated. The standard panel of 24 loci was amplified in a multiplex format (8 triplex PCRs) by using the standardized primers, conditions and loci combinations^6^. PCR amplification products were sized by capillary electrophoresis (3130xl Genetic Analyzer, Applied Biosystems) and assignment of the VNTR alleles was done using Genemapper software (GeneMapper 4.0; Applied Biosystems, Foster City, California, USA).

One case (PrC3) had been diagnosed in Peru before being transferred and was receiving treatment on arrival. The cultures taken at the reception screening were negative. An MTB isolate obtained before he left the Peruvian prison was genotyped.

Another case (PrC2) presented a mixed infection; therefore, MIRU-VNTR was applied on single colonies to determine the MIRU pattern of the two strains present in the coinfection.

### MIRU-VNTR study samples

The MIRU-VNTR patterns obtained were compared with four MIRU-VNTR databases, corresponding to general population from Lima and three international settings receiving Peruvian migrants. The features of these databases were as follows: (i) general population from Lima, Peru: non selected consecutive 370 isolates obtained from the cases diagnosed in the 2010–11 period at the 34 health centers of San Juan de Lurigancho district^7^ and all the MDR isolates (60 isolates) obtained in 2014–15 in the same setting^5^, (ii) Argentina: 404 isolates diagnosed countrywide in 2012–2015 as part of the systematic nationwide surveillance program, which meant >95% of all new MDR-TB cases, (iii) Italy: 87 isolates from Peruvian cases diagnosed in Florence for the period 2001–10^8^ (corresponding to 63% of all the TB cases in Peruvians), together with more than 300 MDR isolates including autochthonous patients and migrants, corresponding to >70% of MDR culture-positive strains diagnosed in Italy, as part of a national MDR surveillance program since 2007^9^ and (iv) Spain: 2890 isolates including autochthonous patients and migrants from a convenience sample. Comparisons of the MIRU-VNTR types were done by using Bionumerics platform (4.6 Applied Maths, Sint-Martens Latern, Belgium).

### Genomic analysis

The DNA used for WGS was purified in Madrid from subcultures on MGIT using a Qiagen kit (QIAamp DNA minikit; Qiagen, Courtaboeuf, France) for transferred inmates and Spanish cases, and from Löwenstein–Jensen cultures using CTAB-based standard purification for Lima cases. Biosafety conditions were guaranteed, performing the first steps in BSL-3 laboratories and moving to BSL-2 laboratories only once mycobacteria were inactivated.

WGS was performed as detailed elsewhere^10^. Briefly, DNA libraries were generated using Nextera XT Library Prep kit (Illumina, San Diego, USA) according to the manufacturer’s protocol, and libraries were run in Miseq or Miniseq devices (Illumina), which generated 35–150 bp paired-end reads and an average per base coverage of 65× (total pair-end reads ranged from 399,973 to 1,795,906 reads, with a percentage of duplicates within them ranging from 0,33 to 1,49%. Percentage of unpaired reads <1%. Percentage of unmapped reads within the total from 0,6 to 8%).

The SNP analysis was performed as described elsewhere^4^. Briefly, we mapped the reads for each strain against the most likely common ancestor of the *M. tuberculosis* complex, which was identical to H37Rv in terms of structure but included the maximum likelihood–inferred ancestral nucleotide positions from a virtual ancestor^11^, by using the Burrows-Wheeler Aligner. SNP calls were made with SAMtools and VarScan (coverage of at least 20×). We kept only the homozygous calls (present in at least 90% of the reads in a specific position) and filtered out potential calling errors by omitting variants detected in repetitive regions, phages, PE/PPE regions, and SNPs close to indels (10 bp window) or in areas with an anomalous accumulation of variants (three or more SNPs in 10 bp). Alignments and SNP variants were visualized and checked with the IGV program. Multiple comparisons between the SNPs from the clustered isolates were made using an in-house script written in R software (R Foundation for Statistical Computing, Vienna, Austria 2011, www.R-project.org).

The median-joining networks were constructed from the SNP matrix generated using NETWORK 5.0.0.1. Median vectors (mv) were defined when the distribution of SNPs indicated the existence of a non-sequenced node corresponding to non-sampled genotype in the cluster. The chronology of acquisition of the SNPs is represented from left to right in the networks.

### Design of a strain-specific PCR based on WGS for the Callao-1 survey

In order to identify specific SNPs for cluster Callao-1, we first filtered the SNPs of this strain with an in-house database, including 381 isolates that underwent high-quality sequencing in our laboratory, and in a second stage with a database which includes 4598 sequenced isolates and is representative of global MTBC diversity^4^. We then selected 3 specific SNPs that were common to all clustered members and 1 specific SNP from the “prison present branch”, which was present in the transferred inmates PrC1 and PrC2 but absent in the other members. The aim of this design was not only to detect new cases infected with Callao-1 strain, but also to determine whether this new case was due to a recent transmission in the prison setting or not. Although the SNPs that were most eligible as targets for Allele Specific Oligonucleotide-PCRs (ASO-PCRs) were those affecting essential genes and producing a synonymous change^12^, only one of the selected SNPs could fulfil both characteristics. The other two common specific SNPs selected were located in essential genes but had to be non-synonymous because of restrictions in primer design. As for the prison present branch–specific SNPs, neither of the two possible SNPs were located in genic regions; therefore, one intergenic region was selected.

The Callao-1 ASO-PCR was designed in a 4-plex single-tube format (Table 1). The conditions were: 1.5 mM MgCl_2_, 0.2 μM of each primer (except Rv3792-Fmut and -R that 0.3 μM is added), 200 μM dNTPs (Roche, Mannheim, Germany), 1x Q-solution, and 0.025 U/μL HotStar Taq DNA Polymerase (HotStarTaq® DNA Polymerase, Qiagen, Hilden, Germany). The PCR conditions were 95 °C for 15 minutes followed by 27–40 cycles of 95 °C for 1 minute, 60 °C for 1 minute and 72 °C for 1 minute and a final elongation step of 72 °C for 10 minutes. The number of cycles was 27 when DNA purified from primary positive cultures and boiled crude extracts from stored frozen isolates were used as a template and 40 when it was DNA purified from sputa. The amplification patterns were analyzed by sizing the amplification products using 1.5% agarose gel electrophoresis. Three different amplification patterns can be obtained depending on whether a new case corresponded to the prison present branch, belonged to the cluster but not to the present branch, or was an infection with a strain other than Callao-1.

**Table 1:** SNPs targeted in the ASO-PCR designed to survey the Callao-1 strain.

Targeted SNP	Primer sequences	PCR product size (bp)	DNA target
A 2365238 G	IG2135-F:5′-CCTTGGCATCTTTGCGATCC-3′	350	All strains except prison present branch variants
	IG2135-Rwt: 5′-GGCAGGCGCAAACGTT-3′		
G 618771 T	Rv0528-F: 5′-GCTGTTCGTGTCCCTCGT-3′	271	Non–Callao-1
	Rv0528-Rwt:5′-GAAGTGGAACACCAGGTTGC-3′		
G 3411528 A	Rv3050c-F: 5′-GTCAGATGCGCCACGAAC-3′	218	Callao-1 (all variants)
	Rv3050c-Rmut: 5′-GCGACGGTACGCACCT-3′		
A 4238842 G	Rv3792-Fmut: 5′-AATCAAGGCGGCGGTAGG-3′	155	Callao-1 (all variants)
	Rv3792-R: 5′-CGTCTGCGGGTAGGTAGTG-3′		

### Expanded analysis of the Callao-1 strain using the strain-specific PCR application

This ASO-PCR was transferred to Peru to be applied *in situ* on a collection of 11 isolates of MDR-TB cases from El Callao prison, which were the only ones available (years 2016–17). The DNA used was purified using GenoLyse kit (Hain-Lifescience, Nehren, Germany) (template DNA concentrations ranged from 0.1 to 1.4 ng/µl).

ASO-PCR was also applied *in situ* on 51 isolates corresponding to a selection of non-genotyped cases from the year 2018 from the Lima general population. Additionally, boiled extracts of all the MDR isolates of Peruvian migrants living in Argentina from 2015 to 2017 (n = 30) and a selection of some representatives of the three main MDR clusters transmitted in Argentina within the Peruvian community (n = 10) were delivered to Madrid to undergo ASO-PCR.

All methods were carried out in accordance with relevant guidelines and regulations. All experimental protocols were approved by the Scientific Committee of Gregorio Marañón University Hospital. Informed consent was obtained from all subjects.

## Results

### Genotypic analysis of MTB strains from transferred inmates

**Figure 1:**
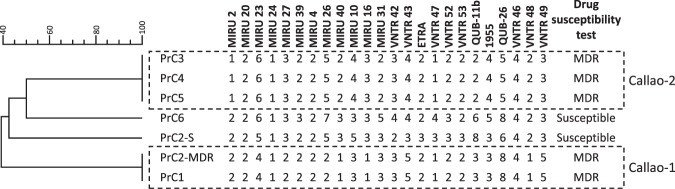
MIRU-VNTR analysis and resistance profile of inmates transferred from El Callao prison to Spain. The two clusters, Callao-1 and Callao-2, are indicated by dashed line rectangles. The MIRU-types for the two strains (MDR and S) coinfecting the patient PrC2 are included.

Since 2016, all inmates transferred from prisons in Peru to complete their sentences in Madrid have undergone systematic health screening. We diagnosed six TB cases at reception. Five had MDR-TB and all from the same Peruvian prison at El Callao district. Their corresponding MTB isolates were genotyped by MIRU-VNTR, revealing that all the MDR cases were clustered (Fig. 1). Two cases transferred from Peru to Spain on two independent flights (case PrC1 in October 2016 and case PrC2 in June 2017) shared the same MDR strain (cluster Callao-1). The other three clustered cases were infected by another MDR strain (Callao-2) and were transferred on two different flights (April 2017 for PrC3 and June 2018 for cases PrC4 and PrC5). The high proportion of clustered cases in such a small sample suggested the presence of two predominant prevalent MDR strains circulating in El Callao prison.

The MIRU-VNTR analysis of the isolate from case PrC2 showed double alleles in 16 independent loci, thus suggesting a mixed infection. The independent analysis of single colonies allowed us to determine the genotype for the accompanying strain and also to determine that it was pansusceptible (Fig. 1). The identification of one mixed infection in a sample of only six cases suggested a likely risk of overexposure due to the high burden of disease in El Callao prison.

### Extended MIRU-VNTR-based analysis of the distribution of the prevalent Callao MDR strains

Given the probable predominance of two MDR strains in El Callao prison, we screened for their presence in other MDR cases from the general population in Lima, Peru, where the prison is located, and also in other countries hosting Peruvian migrants with MDR-TB.

The MIRU-VNTR pattern corresponding to the Callao-1 strain was identified in 4 other MDR-TB cases from the general population of Lima (one case, Lim1, in 2010/11 and 3 cases, Lim2–4, in 2014), but in none of the Peruvian migrants with MDR-TB in Italy and Argentina. The MIRUtype of the Callao-2 strain was identified in another 13 MDR-TB cases in the general population of Lima (5 cases in 2010–11, 7 in 2014, and 1 in 2015), in 3 MDR Peruvian migrants in Italy (years 2009, 2011, 2016), and in 14 MDR cases in Argentina, all living in Buenos Aires area (7 Peruvian migrants, including one who had been in prison in his homeland, and 7 autochthonous cases, during the years 2012–15).

The recent activation of a program to systematically genotype all the resistant isolates in Madrid by MIRU-VNTR led us to draw comparisons with these two strains of interest. We identified the Callao-1 MIRUtype in a recently diagnosed MDR autochthonous case (case MAu1, April 2018). The patient was re-interviewed and informed us that he had been in El Callao prison during 2002–5. This unexpected finding not only confirmed that we were facing a predominant MDR strain, but also that at least for one of the two prevalent El Callao strains corresponded to a long-term, persistant strain (>10 years).

### WGS-based analysis of the Callao-1 strain

**Figure 2:**
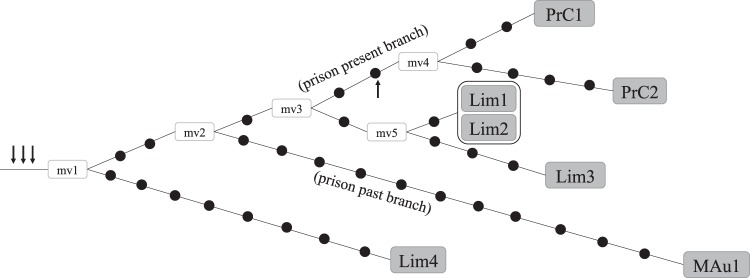
Network of relationships obtained from the WGS analysis of the Callao-1 cluster. Each black dot corresponds to a SNP (details of the SNPs are depicted on the Supplementary Table S1). MV: median vectors. Each box corresponds to a patient. When two or more cases share identical sequences (0 SNPs between them), they are surrounded by a line. Arrows indicate targeted SNPs for the ASO-PCR design.

The fact that one of the prevalent MDR El Callao strains (Callao-1) corresponded to a persistent strain in that setting that had been transferred to the community justified additional characterization and surveying efforts. All the Callao-1 isolates were analyzed by WGS, including the isolates of the two inmates, the ex-convict diagnosed in Madrid and the four cases identified in the general population of Lima. The network of relationships (Fig. 2) showed a distribution of isolates in different branches, with a magnitude of diversity which is consistent with the microevolution expected for a long-term strain. Based on a convenience sample (not derived from systematic population-based genotyping), the presence of several non-sampled nodes (mv) is also expected for our analysis. Cases PrC1 and PrC2 (prison present branch) showed shorter pairwise distances between them than with the other case, MAu1, who had been infected long before in the same prison (prison past branch). The four cases from the general population of Lima were distributed in two different independent branches, three of which (Lim1–3) likely corresponded to direct transmission.

### Optimized screening based on Callao-1-specific PCR

The WGS data revealed the distribution of specific SNPs for the different Callao-1 representatives, thus enabling us to design a multiplex ASO-PCR to survey this strain. Considering the long-term nature of the surveyed strain, the ASO-PCR was designed to do the following: (i) identify new cases infected by any variant of Callao-1 (by means of targeting three SNPs that are common for all seven Callao-1 representatives); and (ii) enable a more detailed analysis of new cases in El Callao by determining whether they corresponded or not to the present branch, which was represented by the PrC cases (i.e., targeting one additional SNP that was shared only by the two PrC representatives, see arrows in Fig. 2).

**Figure 3:**
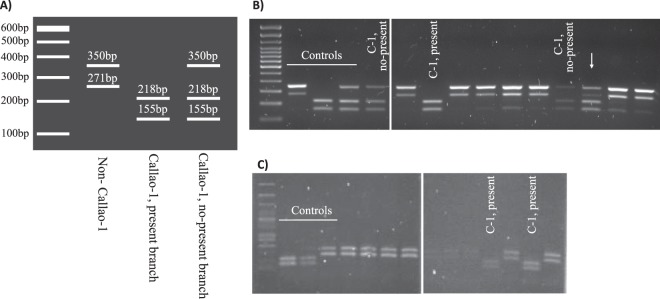
Design and evaluation of the Callao-1 strain–specific PCR. (**A**) *In silico* amplification patterns expected. (**B**) Amplification patterns obtained when applying Callao-1 ASO-PCR to a selection of representatives of the cluster and a random selection of non–Callao-1 strains. The arrow indicates the pattern obtained for a mixed sample (patient PrC2, who presented coinfection with a Callao-1 present branch strain and a non–Callao-1 strain). (**C**) Amplification patterns obtained when applying Callao-1 ASO-PCR to the 11 MDR-TB cases obtained from El Callao prison (years 2016-17). Panels B and C include two parts of the same gel cropped (indicated by a space between them) to eliminate non-informative lanes.

Therefore, we identify three amplification patterns (Fig. 3A) depending on whether the case is infected with (i) a Callao-1 strain, (ii) a variant for the “prison present” branch or (iii) any other strain. The expected patterns for the Callao-1 ASO-PCR were obtained on all the Callao-1 representatives and on a selection of 14 additional non–Callao-1 strains (Fig. 3B).

### Callao-1 MDR screening system based on strain-specific PCR

The final purpose of our strategy was to design a system to optimize the tracking of Callao-1 locally, at El Callao prison, and to extend the surveillance to populations hosting Peruvian migrants.

We evaluated this novel strain-specific PCR strategy on a sample of MDR-TB isolates from Peruvian migrants in Argentina and found that all the isolates corresponded to non–Callao-1 strains.

**Figure 4:**
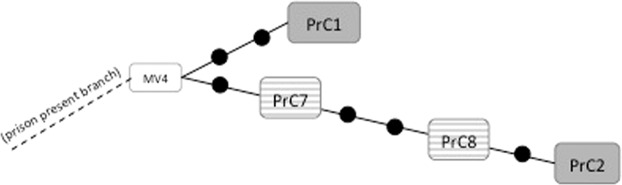
Network of relationships obtained from the WGS analysis of the new isolates, captured by strain-specific PCR (stripped boxes), included in the prison present branch of Callao-1 cluster. The present prison branch corresponds to the one depicted in Fig. 3.

In parallel, the ASO-PCR was transferred to Lima to be applied *in situ* to the latest 11 MDR-TB cases obtained from El Callao prison (years 2016–17) and to non-genotyped cases from the general population of Lima (year 2018). We detected 2 Callao-1 strains within the El Callao prison (Fig. 3C) and none among the general population in Lima. The appropriateness of this assignation was later confirmed by WGS, which located these two isolates in the prison present branch, showing only 1–2 SNPs of distance (Fig. 4), being compatible with recent transmission in the prison.

The remaining nine isolates which lead the non-Callao-1 pattern when applied the strain-specific PCR, were analyzed by MIRU-VNTR. Seven corresponded to the Callao-2 strain; in one case involved in a mixed infection. WGS was performed on these isolates (excluding the mixed infection case) together with the three transferred inmates (PrC3, PrC4 and PrC5), and it revealed short SNP-based distances for most of them (0–3 SNPs pairwise distances) but one, which differed in 11 SNPs with its closest variant.

## Discussion

Molecular epidemiology strategies allow us to identify settings with active transmission of MDR strains. Normally, this requires universal fingerprinting programs, with application of systematic fingerprinting to all the cases in a population to ensure proper capture of transmission clusters. When systematic genotyping is not ensured, missing cases can lead prevalent MDR strains to be overlooked. In our study, we did not have access to systematic fingerprinting data from TB cases in El Callao prison. In fact, we analyzed a very limited sample, comprising only six Spanish inmates transferred from a Peruvian prison to a Spanish prison to complete their sentences. While the size of the sample may seem suboptimal, it was unexpectedly productive on this occasion. Only two different strains (Callao-1 and Callao-2) were responsible for the five cases that proved to be MDR-TB, and one of these cases had a mixed infection. The prevalence of these two strains and the frequency of mixed infections was confimed *in situ* in a more systematic sample of additional cases from the same prison, which revealed that 9 out of 11 MDR cases were infected by either Callao-1 or Callao-2 strains, one of them corresponding to a mixed infection. Our findings highlight the potential magnitude of transmission of MDR and clonally complex infections in El Callao prison.

We found that the magnitude of MDR-TB in the prison was considerable. Even more surprising was the length of time that one of the two MDR strains (Callao-1) was predominant, infecting a patient who had been in the prison more than 10 years previously. Transmission permeability between strains circulating within prisons and the general population^13^ has been reported and can also be deduced from the data in our study. However, as the MIRU-VNTR data available did not correspond to population-based systematic genotyping programs, we cannot quantify precisely the impact of the Callao-1 strain on the general population but only detect their presence, likely favoured by the long time this strain was circulating in that prison. The resulting microevolution led to higher diversity than expected for a short-term recent transmission event, as deduced from the WGS analysis. When we considered all the representative isolates detected for this strain, they were distributed along different branches in the WGS-based network. All the variants (either isolated from cases from prison, from general population of Lima or from the Spanish case likely infected in the prison many years ago) shared an identical MIRU-VNTR pattern, thus rendering this genotyping tool useless for determining whether a new case, sharing that MIRU-VNTR pattern, is due to the active transmission event in the prison or if it corresponds to a variant among those circulating in the general population, or even to a reactivation of the variant which was circulating in the past in the prison setting. The limited value of MIRU-VNTR in complex scenarios, such as the one depicted here, has also been observed in the analysis of clusters involving migrants in Europe, as these also involved several clonal variants sharing identical MIRU-VNTR patterns^2,14,15^. Consequently, WGS was the only way to precisely differentiate between cases due to recent transmission in the host country from those corresponding to independent importations of a prevalent strain in the country of origin.

However, it is not realistic to expect WGS to optimize surveillance of TB in complex epidemiological scenarios worldwide. We propose a shortcut based on tailoring strain-specific PCRs, targeting multiple marker SNPs for the strain to be surveyed taken from the WGS data. In previous studies, we demonstrated the efficiency and versatility of this novel approach for optimizing surveillance of actively transmitted strains^1,16^, fast-tracking secondary cases after importation of XDR-TB^17^, determining the presence and/or prevalence of an outbreak strain in a population by analyzing large collections in remarkable short periods of time^18–20^, and differentiating between recent transmission and importation in clusters involving migrants^4^. Additionally, this strategy has a potential clinical usefulness when tracking resistant strains, as it would allow us to assume the resistance profile of the tracked strain for a new case infected with it, without the need of characterizing specifically the resistance mutations^1,19^. This is because the newly identified cases, who acquired that strain due to recent transmission, must harbour at least the resistance mutations of the parental strain, as these mutations do not revert.

We adapted this approach to prepare a simple tool to simultaneously survey the presence of the Callao-1 MDR strain *in situ* in the Peruvian prison, in the general population of Lima, in three countries hosting Peruvian migrant populations and in all the inmates transferred to Spain. We were thus able to extend coverage of surveillance beyond what would have been possible with standard molecular/genomic strategies. In addition, the sensitivity of strain-specific PCRs has been shown to be sufficient for this approach to be applied directly on stain-positive respiratory specimens^16,17^, thus paving the way for new, real-time surveillance of high-risk strains at diagnosis. Thanks to the application *in situ* of the Callao-1 strain specific-PCR, we were able to reveal two additional cases within El Callao prison, which only difered in 1–2 SNPs from the previous prison cases, confirming the hypothesis of the current transmission event in the prison.

Regarding the other prison strain in study, Callao-2, the analysis of both the transferred inmates to Spain and the eleven isolates from El Callao prison (years 2016–17) indicated that most of the isolates were Callao-2. From the short genome distances obtained after sequencing these isolates it can be deduced that Callao-2 is also actively-transmitted in the prison, which means that only two MDR strains are responsible for nearly all the MDR load in the prison. However, for Callao-2 strain we observed a completely different scenario. According to MIRU- VNTR typing, it was widely distributed in the general population in Lima and in Argentina and Italy, but when analyzing these representatives by WGS a high diversity was observed, markedly superior to the one expected for a recent transmission cluster (up to 37 SNPs of pairwise distance, with 15 SNPs of average pairwise distance; data not shown). This marked diversity makes much more challenging a strain-specific-PCR-based strategy, smilar to the one implemented for Callao-1 strain.

Another relevant finding in our study was the detection, in such a small sample, of two cases (one infected with Callao-1 and the other with Callao-2) showing a mixed infection with a second strain. These findings suggested that clonally complex infections constitute another challenging issue in this setting. In one of the cases we could determine that the MDR strain was accompanied by a pansusceptible strain, which had been initially overlooked at diagnosis. Simultaneous coinfection with a susceptible and a MDR strain could mean a more challenging therapeutical management. Different strains/clonal variants can be found at different anatomic sites in patients with clonally complex infections^10,21^. If something similar is happening in this case and the two different strains were each located in separate cavities, the efficiency of the drugs for reaching the different sites may not be equivalent^22^. The most suitable management of mixed infections involving both resistant and susceptible strains needs to be discussed.

A multinational cooperative effort enabled us to reveal a problematic situation in a Peruvian prison with the coincidence of two prevalent MDR strains, one with a long-term component and likely the cause of frequent mixed infections. The analysis of inmates transferred to Spain provided an indirect opportunity to reveal the features of this pool of complex TB cases, which had also impacted on the general population of Lima and on migrant populations. We must also be alert to the importation of complex MDR-TB cases involving mixed infections. Novel approaches integrating WGS data with the development of strain-specific PCRs could constitute the only means of covering as much as possible the complete international distribution of high-risk strains. Such approaches would provide a more precise snapshot of complex routes involving migrants, inmates, and the general population and thus help us to define more efficient interventions.

## Supplementary information

SupplementaryTable
