# Supplementary material for: Screening of inmates transferred to Spain reveals a Peruvian prison as a reservoir of persistent Mycobacterium tuberculosis MDR strains and mixed infections
Source: Sci Rep. 2020 Feb 17;10:2704. doi: 10.1038/s41598-020-59373-w (PMC7026066; doi:10.1038/s41598-020-59373-w)
Supplement: Supplementary file 1 — SupplementaryTable [file 41598_2020_59373_MOESM1_ESM.pdf]

Suplementary material

"Screening of inmates transferred to Spain reveals a Peruvian prison as a reservoir of persistent Mycobacterium tuberculosis MDR strains and mixed infections."

Estefanía Abascal, Marta Herranz, Fermín Acosta, Juan Agapito, Andrea M. Cabibbe, Johana Monteserin, María Jesús Ruiz Serrano, Paloma Gijón, Francisco Fernández-González, Nuria Lozano, Álvaro Chiner-Oms, Tatiana Cáceres, Pilar Gómez Pintado, Enrique Acín, Eddy Valencia, Patricia Muñoz, Iñaki Comas, Daniela M. Cirillo, Viviana Ritacco, Eduardo Gotuzzo, Darío García de Viedma

Supplementary Table S1. Details of the differential SNPs found in the variants of Callao-1 strain

Position	Ref. allele	Variant allele	PrC1	PrC2	PrC7	PrC8	Lim1	Lim2	Lim3	MAu1	Lim4	Gene name	Essential gene?	Synonymous change?	Function
1175720	C	T	T	C	C	C	C	C	C	C	C	IG1068 (Rv1051c-Rv1052)			
1183195	C	T	T	C	C	C	C	C	C	C	C	Rv1059	No	Yes (Leu>Leu)	hypothetical protein
924872	C	T	C	T	T	T	C	C	C	C	C	IG847 (Rv0831c-Rv0832)			
1259011	C	T	C	T	C	T	C	C	C	C	C	Rv1132	No	No (Gln>Stop)	hypothetical protein
2579208	G	T	G	T	G	T	G	G	G	G	G	IG2341 (Rv2307c-Rv2307A)			
3491767	C	T	C	T	C	C	C	C	C	C	C	IG3176 (Rv3125c-Rv3126c)			
2365238	A	G	G	G	G	G	A	A	A	A	A	IG2135 (Rv2104c-Rv2105)			
3075520	C	T	T	T	T	T	C	C	C	C	C	IG2810 (Rv2765-Rv2766c)			
562874	T	C	T	T	T	T	C	C	T	T	T	Rv0472c	No	No (Asp>Gly)	HTH-type transcriptional regulator
1220204	G	C	G	G	G	G	C	C	C	G	G	IG1111 (Rv1092c-Rv1093)			
1292912	G	C	G	G	G	G	G	G	C	G	G	Rv1163 (narJ)	No	No (Gly>Arg)	respiratory nitrate reductase subunit delta NarJ
1761528	C	T	C	C	C	C	C	C	T	C	C	Rv1556	No	No (Arg>Trp)	HTH-type transcriptional regulator
4215419	G	C	G	G	G	G	G	G	C	G	G	Rv3770c	No	Yes (Gly>Gly)	hypothetical protein
63897	G	T	G	G	G	G	G	G	G	T	G	IG59 (Rv0059-Rv0060)			
310013	G	C	G	G	G	G	G	G	G	C	G	Rv0257	No	No (Trp>Cys)	hypothetical protein
1088782	C	G	C	C	C	C	C	C	C	G	C	Rv0976c	No	No (Ser>Ala)	hypothetical protein
1088783	T	C	T	T	T	T	T	T	T	C	T				

Position	Ref. allele	Variant allele	PrC1	PrC2	PrC7	PrC8	Lim1	Lim2	Lim3	MAu1	Lim4	Gene name	Essential gene?	Synonymous change?	Function
1102878	T	C	T	T	T	T	T	T	T	C	T	Rv0987	No	No (Phe>Leu)	adhesion component transport transmembrane protein ABC transporter
1552588	C	G	C	C	C	C	C	C	C	G	C	Rv1378c	No	No (Ser>Thr)	hypothetical protein
1663350	G	A	G	G	G	G	G	G	G	A	G	Rv1475c (acn)	Yes	Yes (Val>Val)	aconitate hydratase
1931025	A	C	A	A	A	A	A	A	A	C	A	Rv1704c (cycA)	No	No (Ile>Met)	D-serine/alanine/ glycine transporter protein CycA
3232239	T	C	T	T	T	T	T	T	T	C	T	Rv2920c (amt)	No	No (Lys>Glu)	ammonium transporter
3641119	T	C	T	T	T	T	T	T	T	C	T	Rv3261 (fbiA)	No	No (Ser>Pro)	LPPG:FO 2-phospho-L-lactate transferase
4300429	A	G	A	A	A	A	A	A	A	G	A	Rv3826 (fadD23)	No	Yes (Val>Val)	acyl-CoA synthetase
6579	C	A	C	C	C	C	C	C	C	C	A	Rv0005 (gyrB)	Yes	No (Ser>Tyr)	DNA gyrase subunit B (FQ resistance)
507612	G	A	G	G	G	G	G	G	G	G	A	Rv0421c	No	Yes (Ala>Ala)	hypothetical protein
577617	T	G	T	T	T	T	T	T	T	T	G	IG492 (Rv0487-Rv0488)			
1347599	G	A	G	G	G	G	G	G	G	G	A	Rv1204c	Yes	Yes (Ala>Ala)	hypothetical protein
1604091	C	T	C	C	C	C	C	C	C	C	T	Rv1428c	No	Yes (Leu>Leu)	hypothetical protein
2123145	C	T	C	C	C	C	C	C	C	C	T	Rv1872c (lldD2)	No	No (Val>Ile)	L-lactate dehydrogenase (cytochrome) LldD2
4356770	G	A	G	G	G	G	G	G	G	G	A	Rv3878	No	Yes (Gln>Gln)	hypothetical protein
2288859	A	C	D*	D*	D*	D*	A	A	A	A	C	Rv2043c (pncA)	No	No (Val>Gly)	pyrazinamidase/ nicotinamidas PNCA (PZase) (PZA resistance)
2278495	T	G	G	G	G	G	G	G	G	T	T	IG2062 (Rv2030c-Rv2031c)			
365607	A	C	C	C	C	C	C	C	C	C	A	Rv0303	No	No (Asp>Ala)	dehydrogenase/ reductase
815835	T	G	G	G	G	G	G	G	G	G	T	Rv0724 (sppA)	No	No (Leu>Arg)	protease IV SppA

* D: deletion of 15pb (position: 2288853..2288867)
